# Flexural Strengthening of Azobé Hardwood Beams with Externally Bonded CFRP and GFRP Laminates: Experimental Investigation and CNR-DT 201/2005 Assessment

**DOI:** 10.3390/polym18121469

**Published:** 2026-06-11

**Authors:** Ghassan Hachem, Wassim Raphael, Rafic Faddoul

**Affiliations:** Faculty of Engineering and Architecture-ESIB, Saint-Joseph University of Beirut, Beirut 1104 2020, Lebanon

**Keywords:** FRP strengthening, hardwood beams, CFRP, GFRP, marina structures, timber rehabilitation, composite reinforcement

## Abstract

Fiber-reinforced polymer (FRP) composites provide an effective strengthening solution for timber members because of their high tensile capacity, low self-weight, corrosion resistance, and practical applicability in rehabilitation works. Although FRP strengthening of timber beams has been widely investigated, most available experimental evidence concerns softwood and glued-laminated systems, whereas comparatively limited data are available for dense tropical hardwoods used in marine and waterfront infrastructure. This study investigates the flexural behavior of Azobé (*Lophira alata*) hardwood beams strengthened with externally bonded carbon-fiber-reinforced polymer (CFRP) and glass-fiber-reinforced polymer (GFRP) laminates. The main contribution of this work is the application of externally bonded FRP strengthening to Azobé timber members intended for marina pontoon and related waterfront applications, where structural upgrading may be required to accommodate increased service loads. Mechanical characterization of the timber was first conducted through compression and tensile tests. Subsequently, nine beams were tested under three-point bending, including three un-strengthened reference beams, three GFRP-strengthened beams, and three CFRP-strengthened beams. The average ultimate load increased from 26.92 kN for the reference beams to 35.59 kN and 39.85 kN for the GFRP- and CFRP-strengthened beams, respectively. Statistical indicators, including standard deviation, coefficient of variation, standard error, confidence intervals, and two-sample *t*-tests, were included to account for the limited number of specimens and the natural variability of timber. CFRP exhibited the highest mean response within the present test series; however, the difference between the CFRP- and GFRP-strengthened beams is interpreted as an indicative experimental trend rather than a general statistical conclusion. No visible premature de-bonding was observed, and the strengthened specimens failed mainly by FRP rupture, suggesting bond engagement under the tested configuration. Nevertheless, bond behavior was not directly quantified using strain, slip, or interfacial measurements. The experimental results were also compared with analytical predictions based on the Italian guideline CNR-DT 201/2005 and with a simplified section-level interpretation. Overall, the findings indicate that externally bonded FRP laminates can provide a practical upgrading solution for existing Azobé timber members in marina pontoon and waterfront structures, while larger experimental series and direct bond/strain measurements are required for broader validation.

## 1. Introduction and Background

Timber has long been recognized as an efficient structural material because of its favorable strength-to-weight ratio, renewability, ease of construction, and comparatively low environmental impact when obtained from sustainably managed resources. Owing to these advantages, timber beams have been widely used in buildings, bridges, and marine infrastructure, including docks and marina pontoons [1,2,3,4].

**Figure 1:**
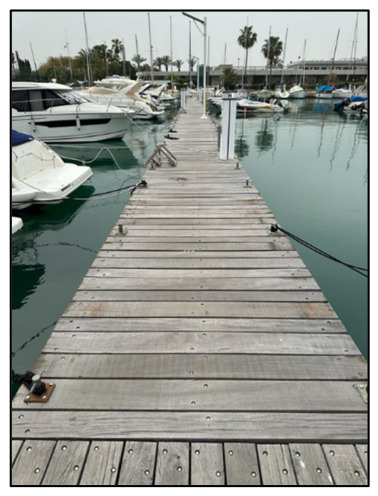
Azobé wood for marina pontoon.

**Figure 2:**
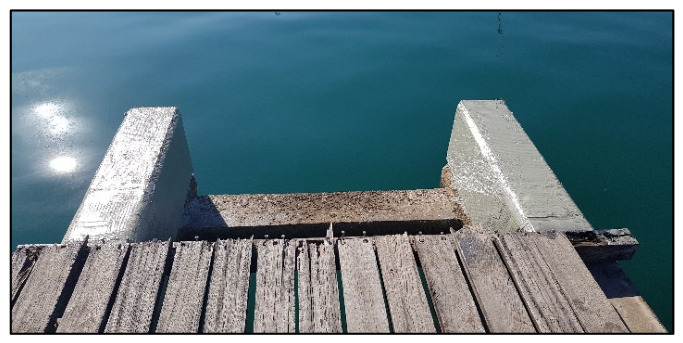
Damaged Azobé wood panels.

Tropical hardwood species such as Azobé (*Lophira alata*) are frequently used in marine and hydraulic environments because of their high density and favorable natural durability. Azobé, in particular, has been associated with resistance to marine biodeterioration and with satisfactory long-term performance in waterfront and sheet-pile applications [5,6,7]. More broadly, the durability and protection of timber structures in marine environments remain critical design considerations, as exposure to moisture, marine organisms, and biological degradation can affect long-term structural performance [5,8]. Representative Azobé elements and deterioration observed in practice are shown in Figure 1 and Figure 2.

Many existing marina structures were originally designed for relatively light service conditions. However, increasing accessibility requirements have introduced localized wheel loads associated with electric mobility scooters and powered wheelchairs used by elderly users. These actions differ from conventional pedestrian loads because they are transferred through relatively small contact areas and may generate elevated local bending stresses in deck planks and supporting beams [9,10,11]. In many cases, the timber members remain structurally serviceable, making strengthening a more economical and environmentally sustainable alternative to complete replacement.

Fiber-reinforced polymer (FRP) composites have emerged as effective strengthening materials because of their high strength-to-weight ratio, corrosion resistance, and suitability for externally bonded reinforcement. In recent years, their application has expanded from established strengthening practice in conventional structural materials to the rehabilitation of timber members [12]. Externally bonded FRP laminates are particularly effective in flexural strengthening because they are positioned in the tensile zone of beams, where they can contribute directly to resisting tensile stresses [12].

Among FRP systems, carbon-fiber-reinforced polymers (CFRP) generally provide high tensile strength, high elastic modulus, and excellent fatigue performance, making them suitable for applications requiring high strengthening efficiency and deformation control. Glass-fiber-reinforced polymers (GFRP), although generally less stiff than CFRP, offer good tensile strength, favorable durability, wide availability, and lower material cost. These characteristics make both CFRP and GFRP relevant for engineering strengthening applications, particularly when improved structural capacity is required without substantially increasing the self-weight of the existing member [12,13].

FRP composites have also been investigated for strengthening and protecting structural materials and infrastructure systems beyond timber. Pultruded GFRP panels have been proposed for the sustainable corrosion protection of steel bulkhead walls in marine environments [14]. Externally bonded CFRP laminates have also been used to strengthen polyethylene storage tanks, demonstrating the potential of FRP systems for polymeric structural components [15]. In addition, CFRP laminates have been applied to marble panels, confirming the effectiveness of externally bonded FRP reinforcement for brittle construction materials [16]. These studies indicate that FRP strengthening is not only limited to conventional concrete or timber systems but can also be adapted to metallic, polymeric, and brittle substrates when appropriate bonding and strengthening configurations are used.

In addition to their mechanical advantages, the long-term durability of FRP composites is a key consideration in engineering strengthening applications, particularly when structural members are exposed to humid, alkaline, or aggressive environments. Yu et al. [17] investigated the durability of carbon–glass hybrid FRP bars exposed to water and alkaline solutions and reported that environmental exposure can affect moisture diffusion and mechanical property retention. Similarly, Xin et al. [18] studied a glass-fiber-reinforced polypropylene cable-anchor component and evaluated its long-term performance under alkaline exposure. Although these studies do not directly address FRP-strengthened timber members, they highlight the importance of durability, environmental resistance, and mechanical retention when CFRP and GFRP materials are used in structural reinforcement.

A substantial body of experimental work has demonstrated the benefits of FRP reinforcement for timber beams. Fiorelli and Dias reported improvements in stiffness and strength for pinewood beams strengthened with externally bonded carbon- and glass-fiber composites [19]. Gentile et al. showed that near-surface-mounted GFRP bars could increase timber-beam flexural strength by approximately 18% to 46% [20], while Alhayek and Svecova found that GFRP laminates increased the strength of reinforced timber beams by approximately 36% on average [21]. Additional studies on CFRP-strengthened solid timber beams confirmed that the structural response depends strongly on strengthening layout and reinforcement length [22,23].

Despite these advances, the available literature remains dominated by studies on softwood species, glued-laminated members, and other conventional engineered timber systems. Recent state-of-the-art reviews similarly emphasize that the experimental database for FRP-strengthened timber is broad for commonly used structural timbers, but remains comparatively limited for tropical hardwood members and for applications involving aggressive exposure conditions [12,13,24,25,26].

For marina pontoon applications, the structural motivation for upgrading existing timber deck members is reinforced by changing service demands. Mobility scooters and powered wheelchairs introduce localized wheel loads over relatively small contact areas. Studies on deck systems under static loading have shown that concentrated loads can generate higher local bending and stress effects than distributed loading patterns, which is directly relevant to deck planks and supporting beams in pontoon systems [9,10,11].

Another important issue for FRP-strengthened timber in marine exposure is the durability of the timber–FRP interface under moisture exposure and environmental cycling. Reviews focused on FRP–wood durability have reported that environmental exposure may degrade the interface and lead to delamination or premature bond-related deterioration if the bonded system is not properly designed. Bonding studies have also shown that adhesive selection, hygrothermal exposure, and strengthening details are critical to the long-term integrity of FRP–wood joints [22,23,24,26,27,28].

Accordingly, there is a clear need for experimental evidence on the flexural strengthening of Azobé hardwood beams using externally bonded CFRP and GFRP laminates, particularly for upgrading existing marina pontoon deck structures. The present study addresses this need by evaluating the flexural response of unstrengthened, GFRP-strengthened, and CFRP-strengthened Azobé beams. The work also compares the strengthening efficiency and observed failure mechanisms of both FRP systems and assesses the experimental results using the analytical framework of CNR-DT 201/2005 [29]. In addition, the study examines the practical feasibility of externally bonded FRP laminates as an upgrading solution for existing Azobé timber members exposed to evolving service demands in marina pontoon and waterfront structures [12,24].

## 2. Materials and Methods

### 2.1. Materials

The experimental program was conducted on Azobé hardwood beams strengthened with externally bonded carbon-fiber-reinforced polymer (CFRP) and glass-fiber-reinforced polymer (GFRP) laminates. Azobé was selected because it is a dense tropical hardwood commonly used in demanding marine and waterfront applications. The strengthening systems consisted of one layer of CFRP laminate and two layers of GFRP laminate bonded to the tension face of the beams using an epoxy adhesive.

**Figure 3:**
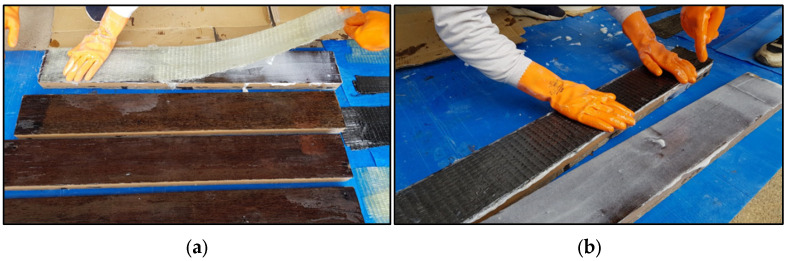
(**a**) Application of GFRP. (**b**) Application of CFRP.

The CFRP strengthening system consisted of LOCTITE Tyfo SCH-41 unidirectional carbon fabric used with LOCTITE Tyfo S Epoxy, whereas the GFRP system consisted of LOCTITE Tyfo SEH-51A unidirectional glass fabric used with the same epoxy adhesive. These systems are produced by Fyfe/Henkel [30] and are commonly used as wet-layup FRP strengthening systems for structural rehabilitation applications. The adopted application procedures for the two reinforcement systems are illustrated in Figure 3a,b.

For the GFRP system, two layers were applied, resulting in a total nominal laminate thickness of 2.6 mm. According to the manufacturer’s data adopted in this study, the CFRP laminate had a nominal thickness of 1.0 mm, a design tensile strength of 903 MPa, and a design elastic modulus of 87 GPa. The GFRP laminate had a nominal thickness of 1.3 mm per layer, a design tensile strength of 455 MPa, and a design elastic modulus of 23.4 GPa. Tyfo S Epoxy is a two-component, ambient-cure matrix material used with Tyfo fabrics to form the wet-layup composite system. Its reported tensile strength, tensile modulus, compressive strength, and compressive modulus are 72.4 MPa, 3.18 GPa, 86.2 MPa, and 3.2 GPa, respectively, as summarized in Table 1. The timber substrate was characterized through compression and tensile testing before the beam-strengthening program.

### 2.2. Preparation of FRP-Strengthened Azobé Beams

Before FRP application, the Azobé beams were visually inspected, and the tension face of each specimen was mechanically prepared to improve the interface between the timber substrate and the composite reinforcement. The bonding surface was sanded along the longitudinal direction of the beam to remove weak surface layers, dust, loose fibers, and local irregularities, while also increasing surface roughness to promote mechanical interlocking with the epoxy adhesive. After sanding, the prepared surface was carefully cleaned to remove loose particles before resin application.

**Figure 4:**
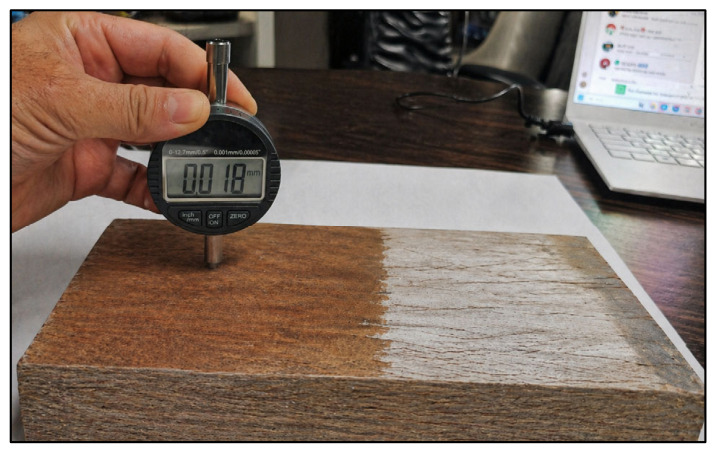
Surface roughness measurement of the mechanically prepared Azobé timber before CFRP application.

To assess the quality of the surface preparation, the roughness of the prepared timber surface was measured before CFRP bonding as a representative evaluation of the treated bonding zone. As shown in Figure 4, the measurement was performed using a digital measuring device, and a representative roughness value of 0.018 mm (18 µm) was recorded. This measurement provided an additional check of the prepared bonding surface beyond visual inspection and supported the intended improvement in adhesive contact and mechanical interlocking. However, it should be noted that this roughness measurement does not constitute a direct quantitative characterization of bond stress transfer or interfacial slip behavior.

**Figure 5:**
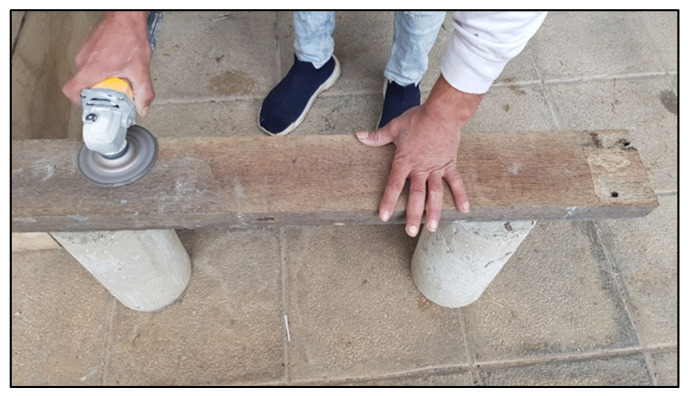
Mechanical grinding of wood beams.

**Figure 6:**
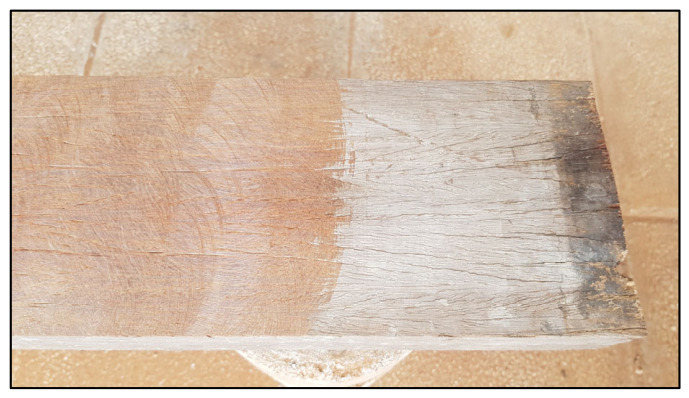
Surface preparation.

**Figure 7:**
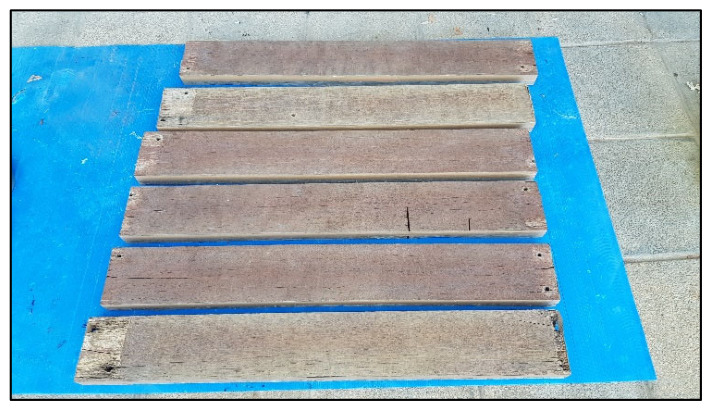
Specimens ready to receive FRP.

The CFRP and GFRP systems were applied using a wet lay-up procedure. The epoxy resin was prepared according to the manufacturer’s recommendations and distributed uniformly over the prepared timber surface. One layer of unidirectional CFRP fabric was applied to the CFRP-strengthened specimens, whereas two layers of unidirectional GFRP fabric were applied to the GFRP-strengthened specimens, as shown in Figure 5, Figure 6 and Figure 7. In all cases, the fibers were oriented parallel to the longitudinal axis of the beam to enhance flexural tensile resistance. The fabrics were impregnated with epoxy, pressed to promote contact with the timber surface, and left to cure under ambient laboratory conditions before testing. After curing, the strengthened specimens were visually inspected to verify the continuity and apparent quality of the bonded reinforcement.

### 2.3. Material Characterization Tests

#### 2.3.1. Compression Tests

**Figure 8:**
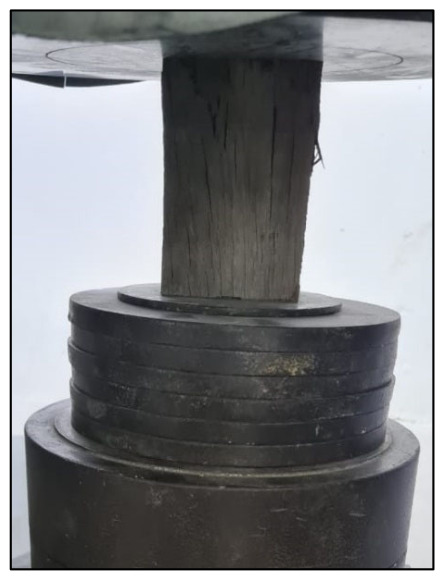
Compression test setup used for the characterization of Azobé timber.

Compression tests were conducted on Azobé wood specimens to characterize the compressive behavior of the timber both parallel and perpendicular to the grain. A total of six compression tests were performed, including three specimens loaded perpendicular to the grain and three specimens loaded parallel to the grain. The test setup is shown in Figure 8.

The compressive strength was calculated by dividing the maximum applied load by the loaded cross-sectional area, as follows:

(1) $$f_{c}=\frac{P_{cmax}}{A}\;$$

where *f_c_* is the compressive strength, *P_cmax_* is the maximum applied load, and *A* is the loaded cross-sectional area.

The recorded load–displacement data were used to calculate the compressive strengths and to generate stress–apparent strain curves for material characterization. Since direct strain-gauge measurements were not available in the laboratory records, apparent strain was calculated by dividing the recorded displacement by the original specimen dimension in the loading direction. Therefore, the resulting curves should be interpreted as stress–apparent strain responses rather than direct local stress–strain measurements. The corresponding curves and measured compressive strength values are presented in the Section 3.

#### 2.3.2. Tensile Tests

**Figure 9:**
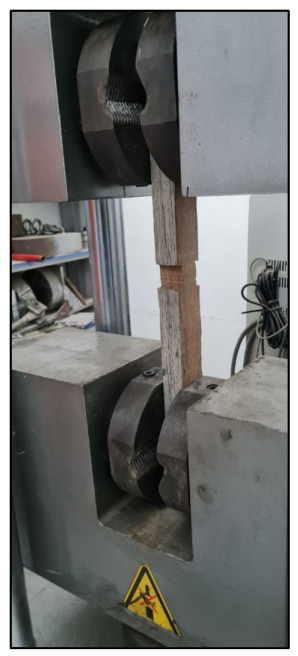
Tension test setup for Azobé specimens tested parallel to the grain.

Tensile tests parallel to the grain were conducted to characterize the tensile resistance of the Azobé timber substrate. This property is relevant to the present strengthening strategy because flexural failure in timber beams is strongly influenced by the tensile behavior of the lower fiber zone. Three specimens were tested in tension parallel to the grain, and the corresponding test setup is shown in Figure 9.

The tensile strength was calculated from the maximum tensile load divided by the net cross-sectional area of the specimen, as follows:

(2) $$f_{t}=\frac{P_{tmax}}{A}$$

where *f_t_* is the tensile strength, *P_tmax_* is the maximum tensile load, and *A* is the net cross-sectional area of the specimen.

The recorded load–displacement data were used to calculate the tensile strengths and to generate stress–apparent strain curves for material characterization. Since direct strain-gauge measurements were not available in the laboratory records, apparent strain was calculated by dividing the recorded displacement by the adopted reference length. Therefore, the resulting curves should be interpreted as stress–apparent strain responses rather than direct local stress–strain measurements. The corresponding stress–apparent strain curves and measured tensile strength values are presented in Section 3.

### 2.4. Beam Specimens and Strengthening Configuration

**Figure 10:**
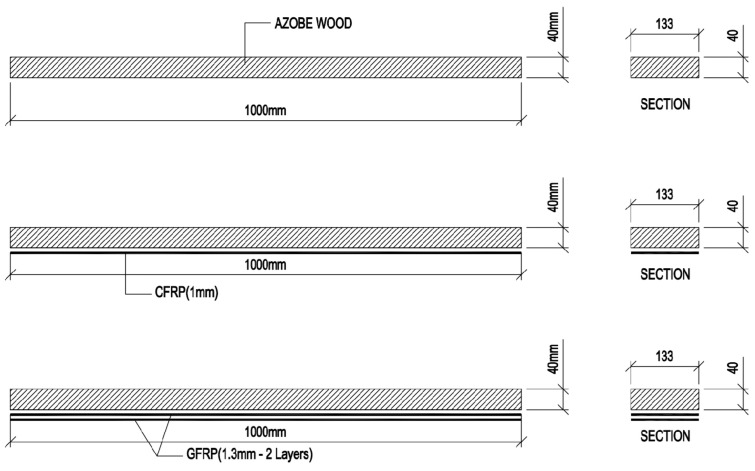
Geometry of reference and FRP-strengthened Azobé beams.

The principal phase of the study consisted of flexural testing of nine Azobé timber beams divided into three groups: three unstrengthened reference beams, three beams strengthened with GFRP laminates, and three beams strengthened with CFRP laminates. All beams had a nominal length of 1000 mm, a clear span of 800 mm, a thickness of 40 mm, and a width of approximately 133 mm. The geometry and strengthening configuration of the reference and FRP-strengthened beams are shown in Figure 10.

**Figure 11:**
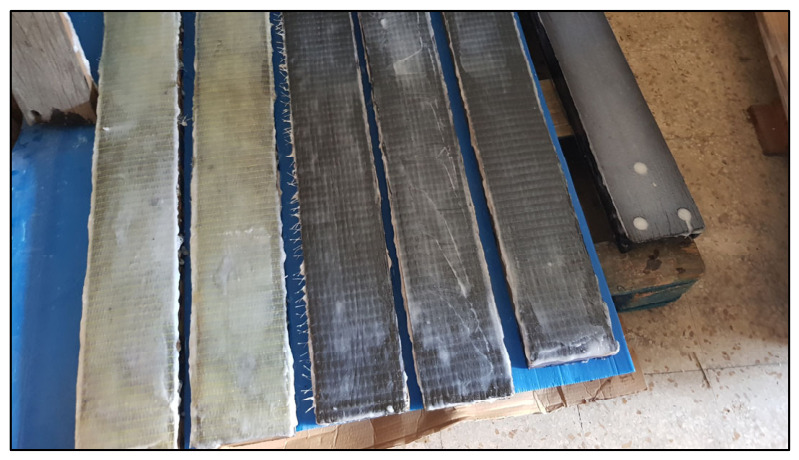
Cured specimens.

The laminates were bonded to the bottom face of the beams, corresponding to the tensile zone under bending. In all strengthened specimens, the FRP fibers were aligned parallel to the longitudinal axis of the beam to improve flexural tensile resistance. The CFRP and GFRP laminates covered the full width of the beam bottom face, as shown in Figure 3 and Figure 11.

The specimen groups were designated as follows: R series for the unstrengthened reference beams, G series for beams strengthened with externally bonded GFRP laminates, and C series for beams strengthened with externally bonded CFRP laminates.

### 2.5. Flexural Test Procedure

**Figure 12:**
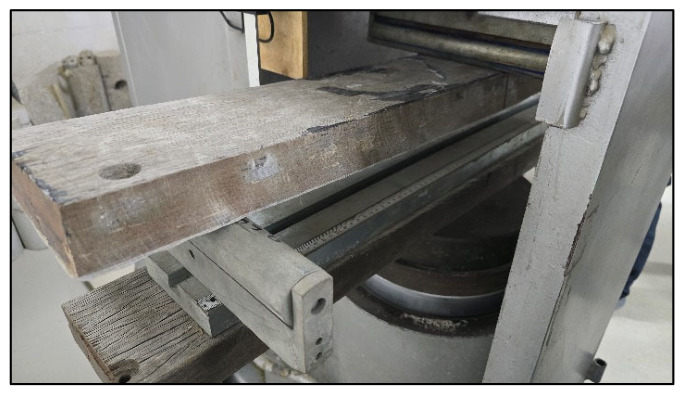
Three points flexural testing setup.

**Figure 13:**
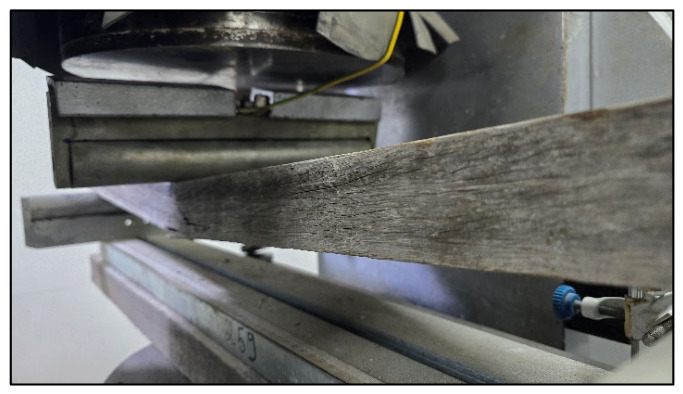
Flexural testing of the reference wood beams.

The flexural performance of the reference and FRP-strengthened Azobé beams was evaluated using three-point bending tests over a clear span of 800 mm. The beams were tested in a simply supported configuration, and the load was applied monotonically at midspan until failure. The corresponding vertical midspan displacement was recorded throughout each test to evaluate the global structural response of the specimens. The test setups are shown in Figure 12 and Figure 13.

The principal measured response was the applied load versus midspan displacement. This response was used to assess the global stiffness, ultimate load, displacement capacity, and failure behavior of the tested beams. The load–displacement curves obtained from the flexural tests are presented and discussed in Section 3.

The strengthening efficiency was calculated using the average ultimate load of each strengthened series relative to the reference series, as follows:

(3) $$\eta =\frac{P_{s}-P_{r}}{P_{r}}\times 100$$

where *η* is the strengthening efficiency, *P_s_* is the average ultimate load of the strengthened series, and *P_r_* is the average ultimate load of the reference series.

## 3. Results and Discussion

### 3.1. Material Characterization of Azobé Timber

The material characterization tests were used to determine the mechanical properties of the Azobé timber adopted in the experimental and analytical interpretation of the strengthened beams. Since the original laboratory records provided load–displacement data without direct strain-gauge measurements, strain values were calculated as apparent engineering strain by dividing the recorded displacement by the original specimen dimension or by the adopted reference length. Therefore, the curves are presented as stress–apparent strain responses rather than direct local stress–strain measurements.

**Figure 14:**
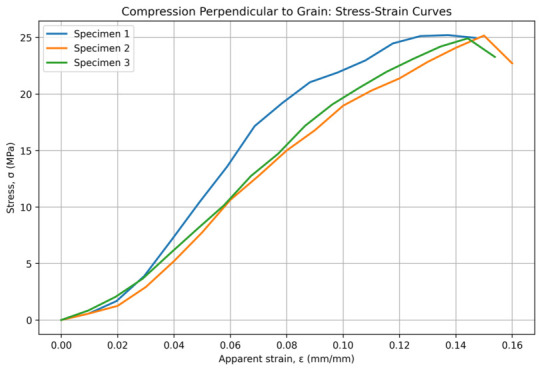
Compression Perpendicular to Grain: Stress–Strain Curves obtained from compression tests performed perpendicular to the grain.

**Figure 15:**
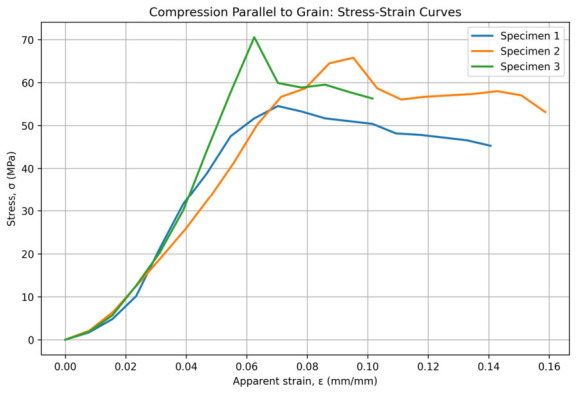
Compression Parallel to Grain obtained from compression tests performed parallel to the grain.

For compression perpendicular to the grain, the measured compressive strengths were 25.2, 25.1, and 24.9 MPa as shown in Figure 14, giving an average value of 25.1 MPa. For compression parallel to the grain, the measured strengths were 54.5, 65.6, and 70.5 MPa as shown in Figure 15, resulting in an average value of 63.5 MPa. These results reflect the anisotropic behavior of Azobé timber and its substantially higher compressive resistance parallel to the grain.

**Figure 16:**
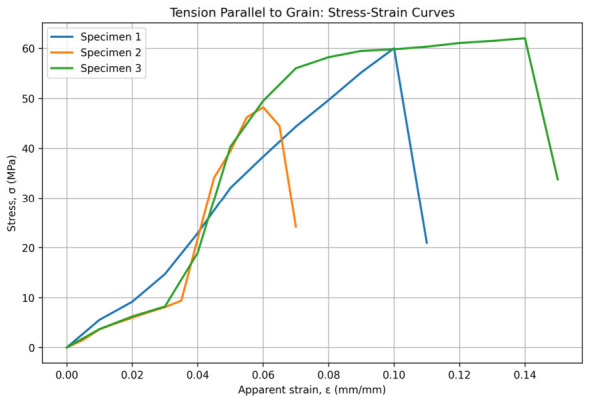
Tension Parallel to Grain: Stress–Strain Curves for Azobé specimens tested parallel to the grain.

The tensile strengths parallel to the grain were 60.0, 48.2, and 62.0 MPa as shown in Figure 16, giving an average value of 56.7 MPa. The corresponding stress–apparent strain curves show noticeable variation among specimens, which is expected for natural hardwood because of anisotropy, local defects, and material heterogeneity. Therefore, the tensile tests were used as supporting material characterization, while the main conclusions of the study were based on the flexural beam tests.

### 3.2. Flexural Behavior of the Reference and Strengthened Beams

**Figure 17:**
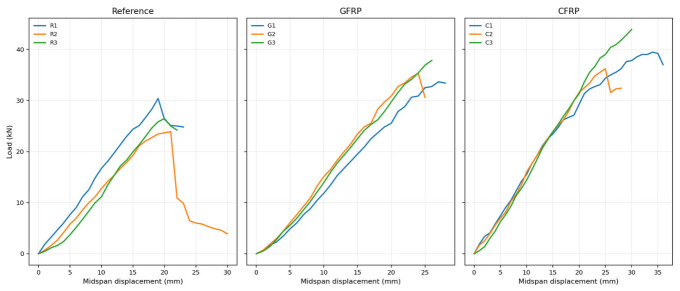
Experimental load–displacement responses of all tested beams, plotted specimen by specimen.

**Figure 18:**
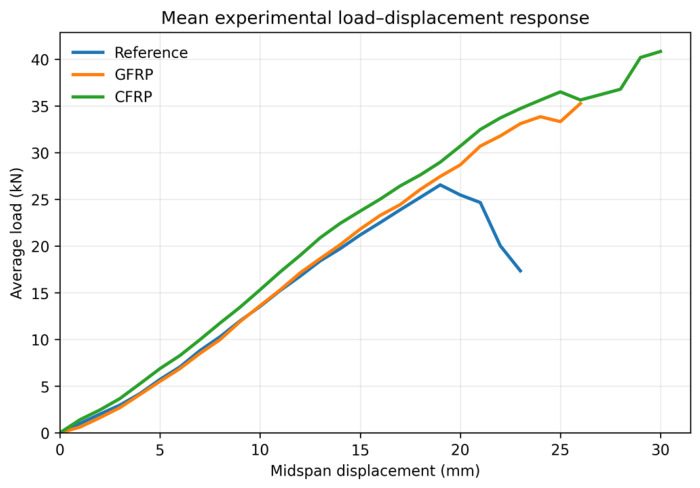
Mean load–displacement curves for the reference, GFRP-strengthened, and CFRP-strengthened beam series.

The flexural tests showed a clear improvement in the global structural response of the Azobé beams after strengthening with externally bonded FRP laminates. The specimen-level load–displacement curves and the corresponding series-average responses are presented in Figure 17 and Figure 18, respectively. These curves show that both FRP systems increased the load carried at a given displacement and improved the ultimate resistance relative to the un-strengthened reference beams.

The reference specimens exhibited the lowest load-carrying capacity and a more limited flexural response compared with the strengthened beams. In contrast, both FRP-strengthened groups showed improved structural performance, indicating the contribution of the externally bonded reinforcement. The reference beams reached ultimate loads of 30.40, 23.90, and 26.46 kN, corresponding to an average ultimate load of 26.92 kN. The GFRP-strengthened beams reached 33.65, 35.30, and 37.83 kN, with an average of 35.59 kN, whereas the CFRP-strengthened beams reached 39.45, 36.21, and 43.90 kN, with an average of 39.85 kN. Relative to the reference series, these values correspond to average strength gains of 32.2% for GFRP and 48.0% for CFRP.

The series-average responses indicate that the strengthening effect was progressively mobilized during loading. CFRP exhibited the highest mean response over most of the displacement range and reached the highest peak load in the present experimental series, while GFRP also provided a substantial increase in resistance. However, because only three specimens were tested per group, the difference between the CFRP- and GFRP-strengthened beams should be interpreted as a sample-level trend within the present dataset rather than as a statistically general ranking.

**Figure 19:**
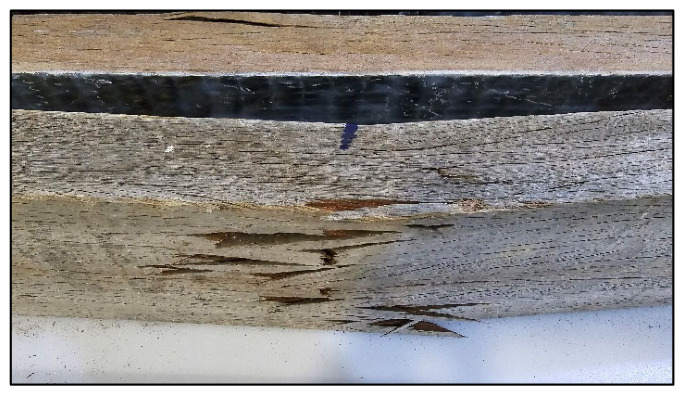
Failure modes observed after flexural testing for the reference beams.

**Figure 20:**
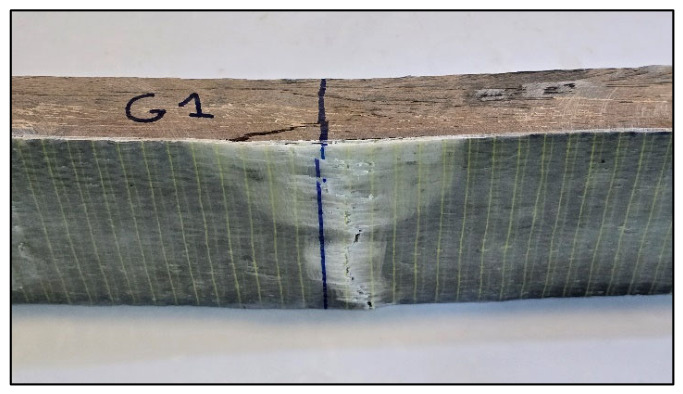
Failure modes observed after flexural testing for the GFRP-strengthened beams.

**Figure 21:**
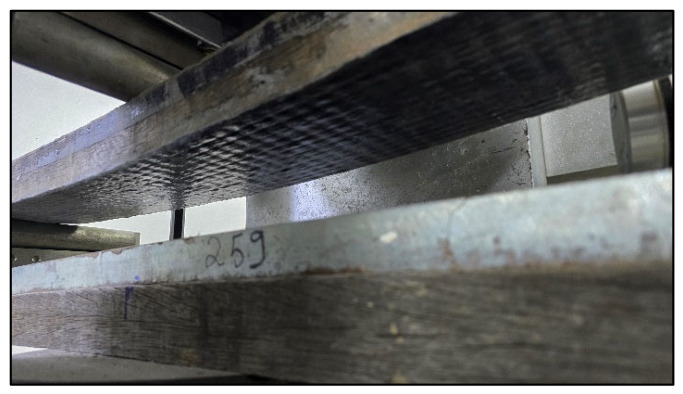
Deflection observed during flexural testing for the CFRP-strengthened beams.

**Figure 22:**
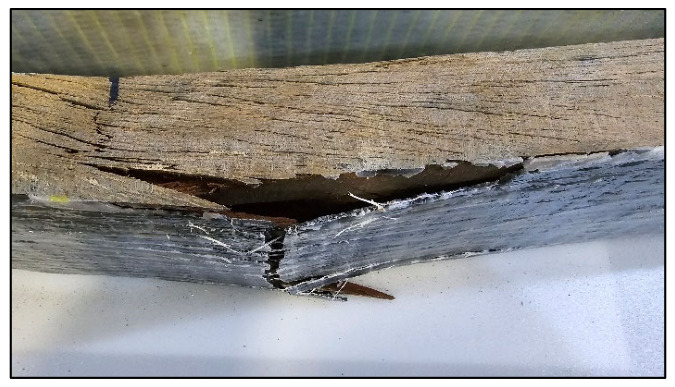
Failure modes observed after flexural testing for the CFRP-strengthened beams.

The strengthened specimens also showed a stiffer global load–displacement response before failure, particularly in the CFRP group. No visible premature debonding was observed during the tests, and failure occurred mainly through tensile rupture of the FRP reinforcement, as shown in Figure 19, Figure 20, Figure 21 and Figure 22. This behavior suggests that the bonded interface was engaged under the specific test configuration. Nevertheless, bond stress distribution, effective bond length, and local interfacial slip were not directly measured. A more detailed discussion of the strengthening mechanism and bond-transfer limitations is provided in the following sections.

To place the present results within the broader context of FRP-strengthened timber beams, the measured strength gains were compared with selected studies from the literature. As summarized in Table 2, the improvement obtained for the GFRP-strengthened Azobé beams falls within the range reported for other timber systems, while the CFRP-strengthened beams showed the highest mean increase within the present experimental program. This comparison supports the potential effectiveness of externally bonded FRP reinforcement for dense Azobé hardwood members; however, the comparison should be interpreted cautiously because of differences in timber species, strengthening configuration, specimen geometry, and experimental conditions among the referenced studies.

### 3.3. Effect of Laminate Type on Flexural Strength and Stiffness Response

Both FRP systems increased the flexural resistance of the Azobé beams, with the CFRP-strengthened specimens showing the highest mean ultimate load within the present test series. This response is consistent with the higher axial stiffness and tensile strength of CFRP, which may allow a larger tensile-force contribution to be mobilized at the bottom face of the beam. However, because no local strain measurements were recorded, the interpretation of strain development, neutral-axis position, and deformation compatibility between timber and FRP remains inferential. Therefore, this discussion should be considered a simplified interpretation based on the global load–displacement response and observed failure modes, rather than a direct experimental validation of the local strain field. The tensile force developed in the FRP laminate may be expressed as:

(4) $$T_{f}\;=\;E_{f}\;A_{f}\;\varepsilon_{f}$$

where *T_f_* is the tensile force developed in the FRP laminate, *E_f_* is the elastic modulus of the laminate, *A_f_* is the laminate cross-sectional area, and *ε_f_* is the tensile strain in the FRP. The corresponding contribution of the laminate to the bending resistance may be expressed as:

(5) $${\Delta M}_{f}\;=\;T_{f}\;z_{f}$$

where Δ*M_f_* is the moment contribution of the laminate and *z_f_* is the distance from the laminate centroid to the neutral axis. Because the FRP is located at the extreme tension face, the laminate benefits from a large lever arm and therefore becomes highly efficient in resisting bending.

For the strengthened beams, the bonded FRP areas were:

(6) $$A_{\left(f,C\right)}\;=\;b\;t_{\left(f,C\right)}\;=\;133\;\times \;1.0\;=\;133\;mm^{2}$$

(7) $$A_{\left(f,G\right)}=b\;t_{\left(f,G\right)}=133\times 2.6=345.8\;mm^{2}$$

Accordingly, the axial stiffness values were approximately:

(8) $$E_{f}\;A_{f}\;\approx \;87,000\;\times \;133\;\approx \;11.6\;\times \;10^{6}\;N\;(CFRP)$$

(9) $$E_{f}\;A_{f}\;\approx \;23,400\times 345.8\;\approx \;8.1\times 10^{6}\;N\;(GFRP)$$

These values indicate that the CFRP reinforcement had a higher axial stiffness than the GFRP reinforcement, even though the GFRP system had a larger bonded area due to the use of two layers. This provides a plausible section-level mechanical interpretation for the higher mean ultimate load observed in the CFRP group within the present experimental dataset. However, the elastic modulus of CFRP was much higher than that of GFRP, whereas the ratio between the average ultimate loads of the CFRP- and GFRP-strengthened beams was only approximately 1.12. This difference indicates that the flexural response was not governed by laminate stiffness alone, but was also affected by strain mobilization, timber heterogeneity, bond engagement, and deformation compatibility limits. Since these mechanisms were not directly measured using local strain or interfacial instrumentation, this interpretation should be considered explanatory rather than experimentally validated at the local level.

The difference between the failure behavior of the CFRP- and GFRP-strengthened beams can be interpreted in relation to the axial stiffness and tensile capacity of the bonded reinforcement. For the same assumed beam curvature, the tensile force mobilized in the FRP layer is expected to increase with *E_f_ A_f_*. Accordingly, the CFRP laminate may mobilize a larger tensile force than the GFRP laminate and may contribute more strongly to the internal resisting moment. This interpretation is consistent with the higher average ultimate load observed in the CFRP-strengthened beams. However, it should not be considered a direct validation of the local strain field because no strain gauges were installed on either the timber substrate or the FRP reinforcement.

**Figure 23:**
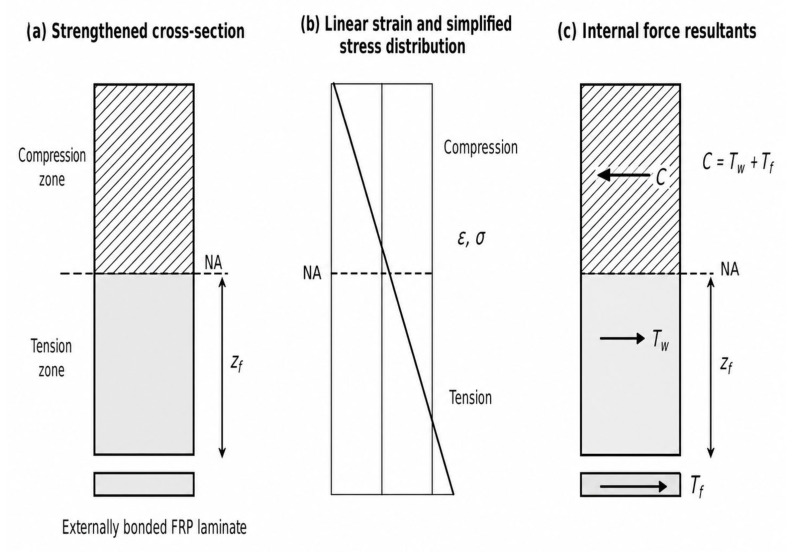
Conceptual strain/stress distribution and internal force resultants in an FRP-strengthened timber beam. The tension-face FRP develops *T_f_* at lever arm *z_f_* relative to the neutral axis, while the timber carries C and *T_w_*.

As illustrated in Figure 23, the strengthened cross-section can be idealized using a linear strain/stress distribution and the corresponding internal force resultants. This representation should be regarded as a simplified section-level model based on plane-section behavior, idealized composite action, and linear response before progressive damage. The externally bonded FRP laminate is positioned near the extreme tension fiber, where it can develop a tensile resultant *T_f_* at a relatively large lever arm *z_f_* with respect to the neutral axis. Because no timber or FRP strain measurements were recorded, the neutral-axis position and strain compatibility assumptions remain analytical assumptions rather than experimentally verified quantities.

In both strengthened groups, no visible premature interface debonding was observed. This observation suggests that the adopted surface preparation and bonding procedure allowed the FRP laminate to remain engaged up to failure under the tested configuration. However, it does not constitute a complete characterization of bond performance, because local interfacial shear stresses, effective bond length, and possible localized slip before failure were not measured. The governing failure mode observed in the tests was FRP tensile rupture rather than global bond separation, which is consistent with a tension-controlled response under the specific short-term monotonic loading conditions adopted in this study.

### 3.4. Variability and Statistical Evaluation of Ultimate Load

As expected for timber members, scatter was observed within each test series because of the natural heterogeneity and anisotropic behavior of wood. To account for the limited number of specimens and to provide a more transparent interpretation of the ultimate-load results, the experimental variability was assessed using the coefficient of variation, standard error, 95% confidence interval of the mean, and Welch two-sample *t*-tests. These statistical indicators were used to support a cautious interpretation of the trends observed within the present dataset, rather than to establish broadly generalizable conclusions. The coefficient of variation was defined as:

(10) $$CoV=\frac{s}{\bar{x}}\times 100$$

where *s* is the sample standard deviation and $\bar{x}$ is the mean ultimate load of the series.

For the reference beams, the mean ultimate load was $\bar{x}$ = (30.40 + 23.90 + 26.46)/3 = 26.92 kN and the sample standard deviation was *s* = 3.27 kN, giving a *CoV* of 12.16%. Using the same procedure, the GFRP-strengthened beams had a mean ultimate load of 35.59 kN and a standard deviation of 2.11 kN, corresponding to a *CoV* of 5.92%. The CFRP-strengthened beams had a mean ultimate load of 39.85 kN and a standard deviation of 3.86 kN, corresponding to a *CoV* of 9.69%.

To improve the transparency of the statistical interpretation of the experimental results, the ultimate load data were evaluated using basic descriptive and inferential statistics. The standard deviation (SD) was used to quantify the scatter of the individual test results around the mean value, while the standard error (SE) was used to estimate the uncertainty associated with the calculated mean. The 95% confidence interval (95% CI) of the mean was then calculated to provide an estimated range within which the true mean ultimate load may lie. Because only three specimens were tested in each group, the confidence intervals are relatively wide and the results should therefore be interpreted with caution, results are gathered in Table 3. These statistical indicators are used to support the comparison between the reference, GFRP-strengthened, and CFRP-strengthened beams within the present experimental dataset, without overgeneralizing the observed trends.

Before comparing the three beam groups, Welch two-sample *t*-tests were performed to evaluate whether the observed differences in ultimate load were statistically supported within the present dataset. Welch’s test was selected because it compares the mean values of two independent groups without assuming equal variances. This assumption is appropriate for timber specimens, where natural variability may differ between the reference, GFRP-strengthened, and CFRP-strengthened beams.

In each comparison, the test examines whether the two groups have the same mean ultimate load. The t-statistic indicates the direction and relative magnitude of the difference between the two means. A negative t-statistic simply indicates that the first group listed in the comparison has a lower mean value than the second group. For example, a negative value for the reference–GFRP comparison is expected because the reference beams had a lower mean ultimate load than the GFRP-strengthened beams.

The *p*-value indicates whether the difference between the two means is statistically significant. A lower *p*-value provides stronger evidence that the two groups are different. The results in Table 4 are therefore used to assess whether the observed increases in ultimate load are statistically supported within the present experimental dataset, while recognizing the important limitation associated with the small sample size.

The statistical results show that both FRP-strengthened groups exhibited higher mean ultimate loads than the unstrengthened reference series. The coefficients of variation were also lower for the strengthened groups within the present dataset. However, because each group contained only three specimens, these findings should be interpreted cautiously as descriptive and statistical trends rather than as population-level validation. Therefore, the results should not be considered proof that FRP strengthening generally reduces timber variability or that CFRP is statistically superior to GFRP for Azobé hardwood members. Larger experimental series are recommended in future studies to improve statistical reliability and to confirm the comparative behavior of the strengthening systems.

### 3.5. Ductility, Stiffness, and Energy Absorption Indicators

To compare the deformation capacity of the three beam series at the group level, a nominal displacement-based ductility index was adopted:

μΔ = δ_u_/δ(0.75u) (11)

where μΔ is the displacement-based ductility index, δ_u_ is the midspan deflection at the peak of the series-average load–displacement curve, and δ(0.75u) is the deflection on the ascending branch of the same mean curve corresponding to 75% of its peak load. This definition was adopted because the timber–FRP beams did not exhibit a distinct yield point.

The resulting values are summarized in Table 5. The series-average values were δ_u_ = 19.00 mm and δ(0.75u) = 14.13 mm for the reference series, δ_u_ = 26.00 mm and δ(0.75u) = 18.28 mm for the GFRP series, and δ_u_ = 30.00 mm and δ(0.75u) = 19.96 mm for the CFRP series.

The calculated ductility indices were μΔ = 1.34 for the reference beams, 1.42 for the GFRP-strengthened beams, and 1.50 for the CFRP-strengthened beams. These values indicate that both strengthening systems improved the nominal deformation capacity relative to the reference series, with the CFRP-strengthened beams showing the highest displacement-based ductility index within the tested dataset.

In addition to the ultimate load increase, quantitative stiffness and energy absorption parameters were estimated from the mean load–displacement curves. Since the original raw acquisition files were not used in this revision, the values reported in Table 6 were obtained from graph-derived characteristic points extracted from Figure 18 and Table 5. Therefore, these values should be interpreted as engineering estimates rather than exact data-acquisition outputs.

The effective ascending stiffness was calculated at 75% of the ultimate load as follows:

K_0.75_ = (0.75P_u_)/Δ_0.75_P_u_ (12)

where K_0.75_ is the effective ascending stiffness, P_u_ is the ultimate load, and Δ_0.75_P_u_ is the displacement corresponding to 75% of the ultimate load.

The secant stiffness at peak load was calculated as:

Ksec,u = P_u_/Δ_u_ (13)

where Ksec,u is the secant stiffness at peak load and Δ_u_ is the displacement corresponding to the ultimate load.

The energy absorption up to peak load was estimated by trapezoidal numerical integration of the mean load–displacement curve between the origin, the point corresponding to 75% of the ultimate load, and the peak-load point:

E_u_ ≈ ½(0.75P_u_)(Δ_0.75_P_u_) + ½(0.75P_u_ + P_u_)(Δ_u_ − Δ_0.75_P_u_) (14)

where E_u_ is the estimated energy absorption up to peak load. The first term represents the area under the curve from the origin to 75% of the ultimate load, while the second term represents the trapezoidal area between 75% of the ultimate load and the peak load.

The results show that the strengthened beams absorbed substantially more energy than the reference beams, mainly because they sustained higher loads over larger displacement ranges. Therefore, the strengthening effect was not limited to peak-load enhancement but also improved the overall structural response in terms of stiffness and energy absorption. However, because the values were derived from plotted curves rather than raw acquisition data, they should be used for comparative interpretation only.

As shown in Table 6, the graph-derived effective ascending stiffness increased from 1.43 kN/mm for the reference series to 1.46 kN/mm and 1.50 kN/mm for the GFRP- and CFRP-strengthened series, respectively. The corresponding estimated energy absorption increased from approximately 257 J for the reference beams to 484 J for the GFRP-strengthened beams and 648 J for the CFRP-strengthened beams. These values correspond to energy ratios of 1.88 and 2.52 for the GFRP and CFRP series, respectively. This comparison suggests that externally bonded FRP laminates increased not only the ultimate load capacity, but also the ability of the beams to sustain load over a larger displacement range. Nevertheless, because the energy and stiffness indicators were extracted from plotted mean curves rather than raw acquisition data, these values should be interpreted as approximate comparative indicators rather than exact experimentally measured material or structural parameters.

### 3.6. Failure Mode and Bond-Transfer Interpretation

A particularly important observation from the beam tests was that no visible premature debonding occurred in the strengthened specimens. Failure occurred mainly through rupture of the FRP laminate at the bottom face. This observation suggests that the bonded reinforcement remained engaged under the tested configuration; however, it should not be interpreted as a complete experimental characterization of the timber–adhesive–FRP interface.

From a mechanical standpoint, the tensile resistance of the strengthened section can be expressed as the combined contribution of the timber and the FRP laminate

(15) $$T=T_{w}+T_{f}$$

where *T_w_* is the tensile force carried by the timber and *T_f_* is the tensile force carried by the FRP.

As the applied load increased, tensile demand developed at the bottom fiber of the beam and was transferred to the FRP through the adhesive layer. Because the laminate was located near the extreme tension face, it was expected to experience high strain demand and to contribute to the tensile resistance of the section. This interpretation is based on classical flexural mechanics, the global load–displacement response, and the observed failure mode; it was not directly verified using local strain gauges.

The stress transfer at the bonded interface may be represented in simplified form using the average bond stress:

(16) $$\tau_{avg}\;=\;\frac{T_{f}}{b_{f}\;l_{b}}$$

where *τ_avg_* is the average bond stress, *b_f_* is the laminate width, and *l_b_* is the bonded length. This expression provides only a simplified average estimate. In externally bonded FRP systems, the actual interfacial shear stress is nonuniform and is usually concentrated near zones of force transfer, cracking, or damage localization. Therefore, the effective bond length may be shorter than the total bonded length, and localized slip may occur even when no global debonding is visible. Since no direct slip measurements, strain profiles, or pull-off/bond tests were performed, the present study cannot quantify the bond-stress distribution, effective bond length, or local interfacial slip. Accordingly, the absence of visible global debonding and the occurrence of FRP rupture only suggest that the interface was sufficiently engaged for the specific beam geometry, surface preparation, laminate layout, and short-term monotonic loading conditions adopted in this study.

The maximum bending moment under three-point bending was calculated as:

(17) $$M_{max}\;=\;\frac{P\;L}{4}$$

Using Equation (17), the average ultimate moments were 5.38 kN·m for the reference beams, 7.12 kN·m for the GFRP-strengthened beams, and 7.97 kN·m for the CFRP-strengthened beams. These moment levels were reached without visible interface debonding, which further supports the interpretation that the bonded reinforcement contributed to the flexural resistance under the tested configuration.

The observed failure mode may therefore be interpreted as consistent with a tension-controlled response in which the externally bonded laminate contributed to the tensile resistance of the section. Nevertheless, this interpretation is based on global load–displacement behavior and post-test failure observations. Strain compatibility, neutral-axis position, bond-stress distribution, and local slip behavior were not directly measured and should therefore be considered assumptions of the simplified mechanical interpretation rather than experimentally verified quantities.

It should be emphasized that the present assessment of bond performance is based on indirect evidence derived from the global structural response and observed failure modes. Consequently, although the absence of visible debonding and the occurrence of FRP rupture suggest effective stress transfer under the adopted test conditions, a detailed characterization of the bond mechanism would require additional instrumentation, such as strain gauges along the laminate, digital image correlation, direct slip monitoring, or dedicated bond tests.

### 3.7. Relevance to Tropical Hardwood Members and Practical Upgrading

The significance of the present results lies in extending the experimental evidence on FRP strengthening beyond conventional softwood and engineered timber systems to a dense tropical hardwood used in marine and waterfront applications. The measured increases in average ultimate load, namely 32.2% for GFRP and 48.0% for CFRP, suggest that externally bonded reinforcement can provide a meaningful flexural-strengthening effect even for a dense substrate such as Azobé.

These increases are structurally relevant for the upgrading of marina pontoon deck members, particularly where localized wheel loads or increased service demands may become critical. The externally bonded bottom-face configuration preserves the original beam geometry, introduces minimal additional self-weight, and can be implemented with less disruption than full replacement. From a practical engineering standpoint, this makes the technique attractive for existing hardwood members that remain serviceable but require enhanced bending capacity.

Nevertheless, the present results should be interpreted as an initial experimental contribution rather than a complete basis for general design recommendations. Further validation under realistic service conditions, including repeated loading, moisture cycling, temperature variation, and marine environmental exposure, is needed before broader design guidance can be established. This is particularly important for waterfront structures, where long-term bond durability and environmental degradation may influence the performance of externally bonded FRP systems.

## 4. Analytical Assessment According to CNR-DT 201/2005

### 4.1. Analytical Framework

The experimental results were compared with the sectional approach proposed in CNR-DT 201/2005 for timber members strengthened with externally bonded FRP laminates. The objective of this comparison was not only to assess the level of agreement between analytical and experimental results, but also to examine the applicability and limitations of the existing design framework when applied to dense tropical hardwood members. The model assumes that plane sections remain plane, that perfect bond is maintained between the timber and FRP reinforcement, that timber behaves linearly in tension and elastoplastically in compression, and that the FRP remains linear-elastic up to failure.

The present analytical assessment was carried out at the critical midspan section under zero axial force:

(18) $$N_{Sd}\;=\;0$$

The flexural resistance was therefore obtained by solving the code equilibrium expressions within the admissible limit regions. A key parameter in the CNR formulation is:

(19) $$k\;=\;\frac{\varepsilon_{cu}}{\varepsilon_{c,el}}\;$$

where *k* is the ratio between the ultimate compressive strain $\varepsilon_{cu}\;$ and the elastic-limit compressive strain $\varepsilon_{c,el}$. Because local compression-strain measurements were not available, the present implementation should be interpreted as an approximate code-based assessment rather than a fully calibrated material model. The parameter *k* was estimated from the compression-test records by assuming that the measured machine displacement represented the specimen shortening. Under this approximation:

(20) $$\varepsilon_{cu}\;\approx \;\frac{\Delta_{u}}{l_{c}}$$

(21) $$\varepsilon_{c,el}\;\approx \;\frac{\Delta_{el}}{l_{c}}$$

(22) $$k\;\approx \;\frac{\Delta_{u}/l_{c}}{\Delta_{el}/l_{c}}=\frac{\Delta_{u}}{\Delta_{el}}$$

where Δ*_u_* is the machine displacement at the ultimate compression load, Δ*_el_* is the displacement at the end of the approximately linear compression range, and *l_c_* is the representative compression gauge length. Using this procedure, the apparent compression parameter adopted in the present implementation was *k* = 9.67. This value should be regarded as an approximate parameter derived from machine-displacement data rather than from direct local strain measurement.

### 4.2. Geometric and Mechanical Parameters

The beam geometry used in the analytical model was B = 133 mm, H = 40 mm, and L = 800 mm. The timber properties adopted were *f_tu_* = 56.7 MPa and *f_cu_* = 63.5 MPa, giving the timber strength ratio *η* = *f_tu_*/*f_cu_* = 0.893. This *η* parameter belongs to the CNR formulation and is distinct from the strengthening efficiency *η* defined earlier in Equation (3).

(23) $$\eta =\frac{f_{tu}}{f_{cu}}=56.7/63.5=0.893$$

(24) $$A_{w}=bh=133\times 40=5320\;mm^{2}$$

(25) $$A_{f,C}=133\times 1.0=133\;mm^{2}$$

(26) $$\rho_{frp,C}=\frac{A_{f,C}}{A_{w}}=133/5320=0.0250$$

(27) $$A_{f,G}=133\times 2.6=345.8\;mm^{2}$$

(28) $$\rho_{frp,G}=\frac{A_{f,G}}{A_{w}}=345.8/5320=0.0650$$

For CFRP, the calculated reinforcement ratio matches the adopted analytical value. For GFRP, the analytical model accounts for the actual two-layer configuration used in the tests, corresponding to a total laminate thickness of 2.6 mm. This gives *Af*,*G* = 345.8 mm^2^ and *ρfrp*,*G* = 0.0650. The modular ratios follow directly from the stiffness contrast between the reinforcement and the timber:

(29) $$n_{C}\;=\;6.73\;n_{G}\;=\;1.81$$

(30) $$p_{frp,C}=0.9875\;p_{frp,G}=0.9675$$

(31) k=9.67

The parameters *p_frp_* remain close to unity in both cases, indicating that the laminates are positioned near the extreme tension fiber. Even with the full two-layer GFRP configuration, *p_frp,G_* = 0.9675, while k continues to influence the neutral-axis depth and therefore the predicted resisting moment.

### 4.3. Admissible Limit Regions

For the adopted material and geometric properties, the admissible solution domains for the strengthened beams correspond to Zones 2, 3, and 4:

(32) $$0\;\leq \;\xi \;\leq \;\frac{1}{1+\eta }$$

(33) $$\frac{1}{1+\eta }\;\leq \;\xi \;\leq \;\frac{k}{k+\eta }$$

(34) $$\frac{k}{k+\eta }\;\leq \;\xi \;\leq \;p_{frp}$$

(35) $$\frac{1}{1+\eta }=0.5283$$

(36) $$\frac{k}{k+\eta }=0.9155$$

**Figure 24:**
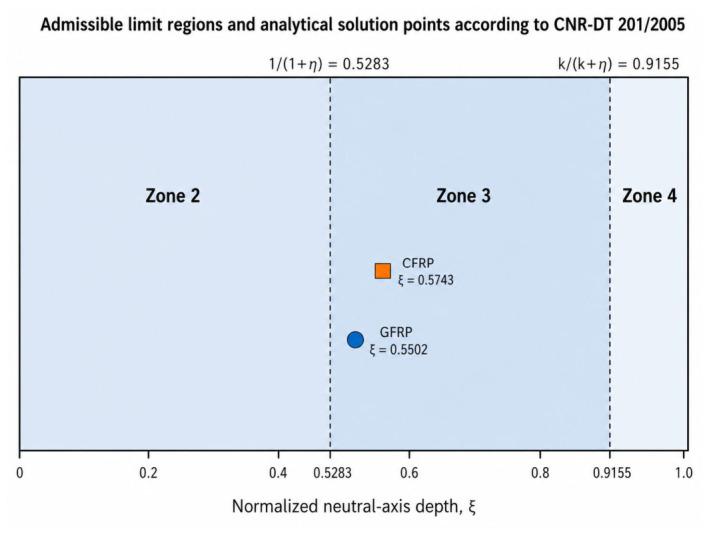
Admissible CNR-DT 201/2005 solution intervals for the normalized neutral-axis depth ξ in the present Azobé–FRP beams. The boundaries are defined by 0, 1/(1 + *η*), k/(k + *η*), *p_frp_*,*G*, *p_frp_,C*, and 1.0.

Figure 24 shows that both strengthened beam configurations satisfy the admissibility requirements of the CNR-DT 201/2005 sectional model and fall within Zone 3. Although both systems are governed by the same analytical regime, the CFRP solution is located slightly farther from the Zone 2/Zone 3 boundary than the two-layer GFRP solution. This position is consistent with the greater stiffness contribution of CFRP in the tension zone. Within the assumptions of the adopted model, this difference helps explain the higher resisting moment and predicted ultimate load obtained for the CFRP-strengthened beams. Overall, the figure supports the internal consistency of the analytical interpretation and clarifies the relative mechanical roles of the two laminate systems, while remaining dependent on the simplifying assumptions of the sectional model.

### 4.4. Governing Analytical Solutions

For both CFRP- and GFRP-strengthened beams, solution of the CNR equilibrium equations produced valid roots in Zone 3:

(37) $$\xi_{C}\;=\;0.5743$$

(38) $$\xi_{G}=0.5502$$

The CFRP configuration yielded a slightly larger neutral-axis depth, whereas the two-layer GFRP configuration also developed a valid Zone 3 solution rather than remaining at the Zone 2/Zone 3 boundary. This reflects the increased contribution of the full GFRP laminate thickness in the analytical model.

### 4.5. Predicted Resisting Moments and Loads

The resisting moment was calculated from the CNR sectional formulation. At an interpretation level, the total resisting moment may also be viewed as the sum of the timber contribution and the FRP contribution, i.e., M = M_wood + M_FRP. In the code-based expression below, M_i(ξ) is the dimensionless resisting-moment function in the relevant admissible zone, ξ is the normalized neutral-axis depth, B is the beam width, H is the beam depth, and f_cu is the timber compressive strength parallel to the grain:

(39) $$M_{Rd}\;=\;M_{i}(\xi )BH^{2}\;f_{cu}$$

For the CFRP series, the predicted resisting moment was 4.42 kN·m, corresponding to a predicted ultimate load of 22.12 kN. For the GFRP series, when the full two-layer laminate configuration was considered, the predicted resisting moment was 3.94 kN·m, corresponding to a predicted ultimate load of 19.72 kN.

(40) M(Rd,C) =4.42 kN·m    P(pred,C) =22.12

(41) $$M_{Rd,G}=3.94\;kN\cdot m\;\;\;\;P_{pred,G}=19.72\;kN$$

### 4.6. Mechanics-Based Load–Displacement Analysis

While the preceding CNR-DT 201/2005 assessment provides a design-oriented estimate of flexural capacity, it does not directly describe the evolution of the load–displacement response. Therefore, a complementary mechanics-based analysis was carried out using transformed-section flexural stiffness. The objective was not to replace the CNR-based strength prediction, but to provide a stiffness-based interpretation of the experimental behavior and to clarify the influence of FRP reinforcement on the initial and post-damage response of the beams.

The analysis was developed using transformed-section flexural stiffness. First, the modulus of elasticity of the Azobé timber was estimated from the initial linear branch of the mean experimental load–displacement curve of the reference beam series. For a simply supported beam under three-point bending, the midspan displacement may be expressed as:

δ = PL^3^/48EI (42)

where P is the applied load, L is the span, and EI is the flexural stiffness of the section. Based on the initial slope of the reference mean curve, the effective modulus of elasticity of the timber was estimated as:

E_w_ ≈ 21.37 GPa

This value was then used in the transformed-section calculations for the three beam configurations.

For the unstrengthened reference beams, the theoretical response was represented by an initial linear elastic branch up to the onset of tensile damage in the bottom timber fibers. The first tensile-damage load was estimated using the elastic flexural stress criterion:

σ_t_ = My/I ≈ f_t_ (43)

where f_t_ = 56.7 MPa is the measured tensile strength parallel to the grain. Using the beam geometry b = 133 mm, h = 40 mm, and L = 800 mm, the theoretical tensile-damage initiation load for the reference beams was found to be:

P_cr,ref_ = 10.05 kN

corresponding to a displacement of approximately:

δ_cr,ref_ = 7.08 mm

For the strengthened beams, the initial uncracked stiffness was determined using the transformed-section method, in which the FRP reinforcement was converted into an equivalent timber area through the modular ratio:

n = E_f_/E_w_ (44)

In this stage, the theoretical initial slopes were:

K_1,ref_ = 1.421 kN/mm

K_1,GFRP_ = 1.743 kN/mm

K_1,CFRP_ = 1.835 kN/mm

These values indicate that both strengthening systems increased the flexural stiffness, with CFRP providing the highest initial stiffness because of its higher elastic modulus.

The first tensile-damage loads of the strengthened beams were also estimated using the transformed uncracked section. The resulting values were:

P_cr,GFRP_ = 13.27 kN

P_cr,CFRP_ = 14.34 kN

corresponding to displacements of:

δ_cr,GFRP_ = 7.61 mm

δ_cr,CFRP_ = 7.82 mm

respectively. These values show that the FRP laminates delayed the onset of tensile damage in the timber tension zone.

After tensile damage initiation, a second theoretical branch was derived for the strengthened beams using a cracked transformed section, in which the tensile contribution of the damaged timber zone was neglected while the FRP laminate remained active in tension. This resulted in reduced but still significant post-damage stiffness values of:

K_2,GFRP_ = 0.803 kN/mm

K_2,CFRP_ = 1.004 kN/mm

The higher post-damage stiffness of the CFRP-strengthened beams is consistent with the greater axial stiffness of the CFRP laminate and provides a simplified mechanical explanation for the higher mean response observed experimentally. However, because no local strain measurements were recorded, this interpretation remains analytical and should not be considered a direct experimental validation of strain compatibility or neutral-axis position.

**Figure 25:**
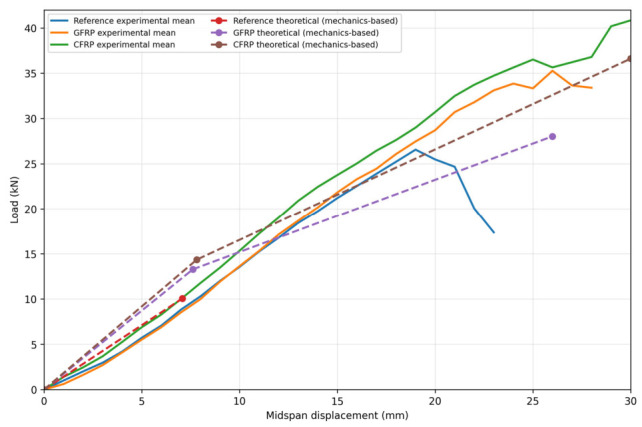
Comparison between the experimental mean load–displacement curves and the mechanics-based theoretical curves for the reference, GFRP-strengthened, and CFRP-strengthened Azobé beams, based on transformed-section flexural stiffness analysis.

Figure 25 compares the experimental mean load–displacement curves with the corresponding mechanics-based theoretical curves. The theoretical model reproduced the expected ranking of the three beam systems, namely CFRP > GFRP > reference, in terms of idealized initial stiffness and resistance to tensile-damage initiation. The analysis suggests that both FRP systems improved stiffness and delayed damage initiation, while CFRP was more effective in maintaining stiffness after tensile damage had begun. This trend is consistent with the experimental observations, but it remains based on simplified transformed-section assumptions rather than direct strain-field validation.

It should be noted that the mechanics-based model is intended as a simplified interpretation of the global structural response rather than a full nonlinear constitutive simulation. The main assumptions of the model include plane-section behavior, idealized composite action, linear response before damage progression, and neglect of local interfacial slip. The reference beam was represented only up to the tensile-damage initiation stage, because a reliable extension of the post-damage branch would require an explicit tension-softening law for timber. For the strengthened beams, the bilinear representation also remains an approximation.

A sensitivity review indicates that the predicted response is influenced by the assumed timber modulus, FRP modulus, laminate area, bond engagement, and post-damage stiffness idealization. Therefore, the model provides a physically meaningful interpretation of stiffness evolution within the adopted assumptions, but it should not be considered an experimentally validated representation of the local strain field or damage progression.

The mechanics-based interpretation presented above provides a stiffness-oriented explanation of the response development. The following subsection compares the analytical predictions with the experimental results in terms of global structural performance.

### 4.7. Comparison with the Experiments

**Figure 26:**
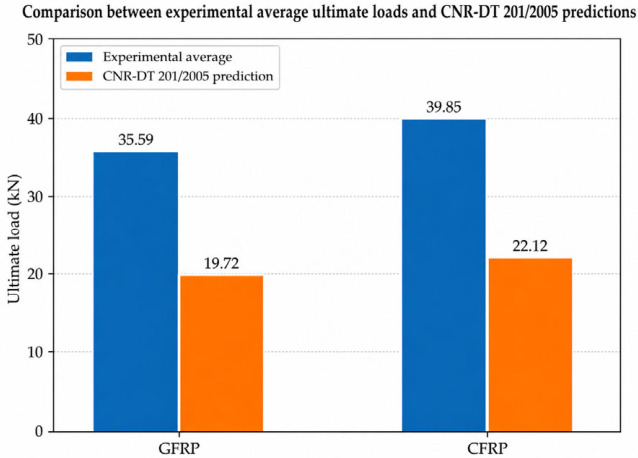
Comparison between the experimental average ultimate loads and the analytical predictions based on CNR-DT 201/2005. The analytical approach remained conservative for both strengthening systems.

A direct comparison between the experimental averages and the CNR-DT 201/2005 predictions is presented in Figure 26. For the GFRP beams, the code-based analytical model predicted an ultimate load of 19.72 kN, whereas the corresponding experimental average was 35.59 kN. For the CFRP beams, the predicted ultimate load was 22.12 kN, compared with an experimental average of 39.85 kN.

(45) $$\frac{P_{exp,G}}{P_{pred,G}}=35.59/19.72=1.80$$

(46) $$\frac{P_{exp,C}}{P_{pred,C}}=39.85/22.12=1.80$$

These ratios show that the present CNR implementation remained conservative for both strengthening systems. The magnitude of underestimation is comparable for GFRP and CFRP, indicating that the analytical model captured the relative ranking of the two laminates more reliably than the absolute capacity levels.

### 4.8. Discussion of the Analytical–Experimental Discrepancy

The analytical results showed a conservative bias when compared with the experimental response. This discrepancy can be explained by the different purposes and assumptions of the analytical approaches used in this study. The mechanics-based transformed-section analysis was primarily intended to interpret stiffness evolution, tensile-damage initiation, and the staged response of the beams. In contrast, the CNR-DT 201/2005 assessment was used as a design-oriented prediction of flexural resistance.

The mechanics-based load–displacement analysis reproduced the expected qualitative response hierarchy among the three beam series. The CFRP-strengthened beams showed the highest theoretical initial and post-damage stiffness, followed by the GFRP-strengthened beams and then the unstrengthened reference beams. This trend is consistent with the experimental results and supports the interpretation that the higher axial stiffness of CFRP contributed to the improved global load–displacement response. However, this model remains a simplified interpretation because it is based on transformed-section stiffness and does not explicitly include nonlinear timber damage, local crack development, interfacial slip, progressive bond degradation, or direct strain-field validation.

In addition, the experimental failure mode was governed mainly by FRP rupture without visible premature global debonding. This observation suggests that the timber–adhesive–FRP interface was engaged under the tested conditions. Nevertheless, local bond behavior, strain transfer, and possible slip development were not directly quantified.

The model captured the relative ranking of the two laminate systems, with CFRP providing higher predicted and mean experimental capacity than GFRP within the tested specimens. However, this comparison should be interpreted cautiously because the CFRP–GFRP difference was not statistically conclusive within the limited dataset.

Overall, the analytical–experimental comparison indicates that the two approaches provide complementary information. The mechanics-based analysis offers a simplified explanation of stiffness development, tensile-damage initiation, and post-damage response, whereas the CNR-DT 201/2005 model provides a conservative estimate of flexural capacity. The remaining discrepancy highlights the need for future calibration using direct strain measurements, interfacial slip monitoring, dedicated bond tests, and larger experimental datasets.

## 5. Conclusions

This study investigated the flexural strengthening of Azobé (*Lophira alata*) timber beams using externally bonded GFRP and CFRP laminates. Both FRP systems improved the global structural response of the tested beams. The average ultimate load increased from 26.92 kN for the unstrengthened reference beams to 35.59 kN for the GFRP-strengthened beams and 39.85 kN for the CFRP-strengthened beams, corresponding to mean gains of 32.2% and 48.0%, respectively. The statistical evaluation indicated meaningful increases for both strengthened groups relative to the reference group within the limited experimental dataset. However, the difference between the GFRP- and CFRP-strengthened beams was not statistically conclusive. Therefore, the higher mean response of CFRP should be interpreted as an experimental trend for the present specimens rather than as a general comparative conclusion.

Among the two strengthening systems, CFRP showed the highest mean strengthening efficiency in the present experimental series, which is consistent with its higher tensile capacity and axial stiffness. Both GFRP and CFRP improved the nominal deformation capacity of the beams, with the CFRP-strengthened series showing the highest displacement-based ductility index. Graph-derived stiffness and energy calculations also indicated that the strengthened beams absorbed more energy up to peak load than the reference beams. However, these values should be interpreted as approximate engineering estimates derived from the load–displacement curves rather than exact data-acquisition outputs.

The observed failure behavior supports the practical potential of the adopted strengthening technique under the tested configuration. No visible premature global debonding was observed, and failure occurred mainly through rupture of the FRP laminate at the tension face. This observation suggests that the timber–adhesive–FRP interface was engaged during the tests. Nevertheless, bond behavior was not directly quantified; therefore, this observation should not be interpreted as general proof of satisfactory bond performance. Future studies should include direct bond characterization, interfacial slip monitoring, and local strain measurements.

The analytical assessment provided complementary insight into the experimental behavior. The mechanics-based transformed-section analysis offered a simplified explanation of the observed stiffness hierarchy and suggested that the FRP laminates contributed to delaying tensile-damage initiation in the timber tension zone, with CFRP providing the highest idealized post-damage stiffness. However, this mechanics-based interpretation was not experimentally validated by local timber or FRP strain measurements. The CNR-DT 201/2005 comparison reproduced the general tension-controlled nature of the strengthened response, but the CNR-based predictions remained conservative, mainly because of uncertainties associated with the timber compression parameter k, which was estimated from machine displacement rather than direct local strain measurements. Accordingly, the analytical assessment should be regarded as a useful conservative comparative tool rather than a fully calibrated prediction model for Azobé hardwood beams.

Overall, the findings indicate that externally bonded FRP laminates have strong potential for the rehabilitation and upgrading of existing Azobé structural members used in marina pontoons and waterfront structures. However, the present work should be interpreted as a proof-of-concept experimental investigation and preliminary analytical assessment, since it was based on a limited number of specimens, one timber species, one beam geometry, and short-term monotonic loading. Future research should include larger test series, additional strengthening configurations, direct local strain instrumentation, dedicated bond tests, statistically robust comparative evaluation, and long-term durability assessments under realistic service conditions, including creep, fatigue, cyclic loading, wet–dry exposure, saltwater action, and hygrothermal conditioning.

## Figures and Tables

**Table 1:** Mechanical properties of the FRP systems and epoxy adhesive used in the study.

Material/System	Source/Manufacturer	Thickness	Tensile Strength	Tensile/Elastic Modulus	Elongation
CFRP laminate	LOCTITE Tyfo SCH-41, Fyfe/Henkel	1.0 mm	903 MPa	87 GPa	0.90%
GFRP laminate	LOCTITE Tyfo SEH-51A, Fyfe/Henkel	1.3 mm/layer; 2.6 mm total	455 MPa	23.4 GPa	1.80%
Epoxy adhesive/matrix	LOCTITE Tyfo S Epoxy, Fyfe/Henkel	—	72.4 MPa	3.18 GPa	5.00%

The properties listed in Table 1 were obtained from the manufacturer’s technical data sheets [30].

**Table 2:** Comparison with literature.

Study	Timber Type	FRP Type	Strength Increase
Gentile et al. [20]	Softwood	GFRP	18–46%
Alhayek & Svecova [21]	Timber	GFRP	~36%
Present study	Azobé hardwood	GFRP	32.20%
Present study	Azobé hardwood	CFRP	48.00%

**Table 3:** Descriptive statistical parameters of ultimate load for the tested beam series.

Series	Ultimate Loads (kN)	Mean Pu (kN)	SD (kN)	*CoV* (%)	SE (kN)	95% CI of Mean (kN)
Reference	30.40, 23.90, 26.46	26.92	3.27	12.16	1.89	18.79–35.05
GFRP	33.65, 35.30, 37.83	35.59	2.11	5.92	1.22	30.36–40.82
CFRP	39.45, 36.21, 43.90	39.85	3.86	9.69	2.23	30.26–49.44

**Table 4:** Welch two-sample *t*-test results for ultimate-load comparisons.

Comparison	t-Statistic	*p*-Value	Interpretation
Reference vs. GFRP	−3.86	0.024	Significant increase vs. reference
Reference vs. CFRP	−4.43	0.012	Significant increase vs. reference
GFRP vs. CFRP	−1.68	0.189	Not statistically conclusive

**Table 5:** Displacement-based ductility indices of the tested beam series.

Series	δ(0.75u) (mm)	δ_u_ (mm)	μΔ [−]
Reference	14.13	19.00	1.34
GFRP	18.28	26.00	1.42
CFRP	19.96	30.00	1.50

**Table 6:** Graph-derived stiffness and energy absorption parameters of the mean load–displacement curves.

Series	P_u_ (kN)	Δ_0.75_P_u_ (mm)	Δ_u_ (mm)	K_0.75_ (kN/mm)	Ksec,u (kN/mm)	Energy to P_u_ (J)	Energy Ratio
Reference	26.92	14.13	19.00	1.43	1.42	257	1.00
GFRP	35.59	18.28	26.00	1.46	1.37	484	1.88
CFRP	39.85	19.96	30.00	1.50	1.33	648	2.52

## Data Availability

The data supporting the reported findings are contained within the article. Additional specimen-level test data and calculation records may be made available by the corresponding author upon reasonable request.
